# Pairwise Diverse and Uncertain Gradient-Sampling for Similarity Retrieval

**DOI:** 10.3390/s25226899

**Published:** 2025-11-12

**Authors:** Christoffer Löffler

**Affiliations:** Escuela de Ingeniería Informática, Pontificia Universidad Católica de Valparaíso, Valparaíso 2340025, Chile; christoffer.loffler@pucv.cl

**Keywords:** machine learning, representation learning, information retrieval, active sampling, position data

## Abstract

**Highlights:**

**What are the main findings?**

- PairPUG selects the most informative training pairs using gradient-based feedback, reducing computation in trajectory representation learning.
- On basketball and American football trajectory datasets, PairPUG halves training time while maintaining or improving retrieval accuracy.

**What is the implication of the main finding?**

- PairPUG provides a scalable and efficient solution for pairwise distance learning on large, unstructured trajectory datasets.
- The approach is generalizable beyond soccer analytics to basketball and American football, and potentially to similar trajectory-heavy domains.

**Abstract:**

Sports tracking produces large, unstructured trajectory datasets. The search and retrieval of interesting plays are essential parts of their analysis. Since annotations are sparse, similarity search remains the standard technique. It relies on learned lower-dimensional representations for its computational feasibility. Siamese Networks learn dimensionality reduction from pairwise distances. However, complete training datasets are impractical to compute due to their combinatorial nature and the cost of distance calculations. Sub-sampling sacrifices representation quality for speed, leading to less meaningful search results. We propose the novel sampling technique Pairwise Diverse and Uncertain Gradient (PairDUG), which exploits the model’s gradient signals to select representative and informative pairs for training. The broad experimental study implements the method for large-scale basketball and American football datasets. The results show that PairDUG at least halves the required compute time while maintaining, or even improving, retrieval quality, and outperforms other baseline methods. Furthermore, our evaluation shows that the selected pairs’ gradient signals exhibit greater magnitude, diversity, and stability than those of any other method. This work represents a foundational contribution to pairwise distance learning. Hence, future work transfers the method not only to other sports, such as soccer, but also to complex trajectory datasets outside the sports domain.

## 1. Introduction

The widespread availability of position-tracking systems across many industries has enabled innovations in trajectory mining. Sports [1,2,3] and operations research [4,5,6] in particular benefit from these large datasets of unstructured multi-trajectory time series. An important application is the search and retrieval of similar instances based on similarity metrics [1,7,8,9]. Due to the scale of the datasets, a complete pairwise comparison of high-dimensional data is infeasible. Previous solutions either filtered the data [1,8,9] or trained Siamese Networks to learn lower-dimensional embeddings that permit efficient search [2,7].

However, filtering datasets reduces the effective search space, resulting in inferior results. On the other hand, learning an embedding of sufficiently high quality for meaningful retrieval results is extremely costly: first, an optimal assignment between two instances’ trajectories is computed using the Hungarian algorithm, and second, a suitable distance metric is selected, such as the Euclidean distance. These pairwise comparisons lead to a significant computational complexity of $O(N^{3})$ for *N* samples in the training dataset.

Sub-sampling of pairs of trajectories is the standard practice for sports scene search [2]. However, while such random sampling reduces the training cost considerably, it leads to an impoverished quality of the embedding space, because it samples fewer pairs, which tend to be less meaningful and easier. Random sampling lacks a mechanism to select more informative or representative data.

In this work, we propose a novel active sub-sampling technique that exploits gradient signals to select pairs that produce diverse gradients with high magnitudes, which we call Pairwise Diverse and Uncertain Gradient (PairDUG). The sampler is inspired by Active Learning [10], specifically BADGE [11], which constructs sets of query instances that are both representative and informative to reduce annotation cost in classification. We transfer the concept to pairwise similarity learning.

We pose three research questions: RQ_1_: Can the required compute time for training a Siamese Network be reduced while maintaining similar retrieval quality? RQ_2_: Does active sampling retain a higher retrieval quality compared to other baselines? RQ_3_: What explanations for the active samplers’ effects on learning are there? Our experiments then determine which samplers achieve the highest retrieval quality and calculate an efficiency score based on runtime and retained performance relative to baselines, such as “simulated full training” and random sampling.

We summarize our contributions as follows:

- We transfer similarity learning from soccer [2] to American football [12] and basketball [13] datasets.
- We propose the novel Pairwise Diverse and Uncertain Gradient (PairDUG) method that extends BADGE sampling [11] to pairwise metric learning via proxy distance cost generated using uniform keypoint sub-sampling. This sampler increases the efficiency of Siamese Network training by sampling the most relevant pairs, selecting batches of instances with diverse and informative gradient signals.
- We experimentally analyze the quality of the learned embeddings on two large-scale real-world datasets, including baseline heuristics such as semi-hard sampling [14] and active samplers such as Entropy [15] and MC Dropout [16] uncertainty-based sampling, and CoreSet [17] diversity-based sampling.
- We release the code and models for reproducing our results (code and models available at https://github.com/crispchris/Pairwise-Diverse-and-Uncertain-Gradient-Sampling-for-Similarity-Retrieval, accessed on 10 November 2025).

## 2. Related Work

The related work for this project spans several distinct fields, each contributing to specific parts of the project. First, the application of the algorithms developed in this paper is the retrieval of sports scenes from large tracking datasets, as shown in Section 2.1. Second, Section 2.2 provides an overview of mining informative data for training Deep Neural Networks. Lastly, we describe samplers from the field of Active Learning in Section 2.3, as we transfer key components of these samplers to this application.

### 2.1. Sport Scene Search

In the application of sports scene search, retrieval methods are classified into two approaches. A family of search methods relies on event data—i.e., annotations or metadata—to retrieve plays. To this end, specialized SQL dialects [18] or graphical query languages [19] facilitate search. However, annotations are typically sparse and costly to procure. The second type of approach directly processes trajectory data using similarity metrics between instances of sports scenes. These methods’ limitations lie in the heuristics they employ to assign sub-trajectories to resolve the similarity computation. These heuristics can be fine-grained and specific to matches, teams, or players [1], and do not generalize well across sports domains. Others are limited by optimizing computational cost via hierarchical templates that reduce the search space for similarity searches [20]. In this regard, pre-clustering of the search space via k-means [20] or ranking [9] can reduce complexity at the cost of retrieval quality.

In an attempt to combine the advantages of the interpretability of event data annotations with similarity learning, Wang et al. [21] segment scenes into semantic chunks, e.g., ball possession, which allows a more natural search at the cost of flexibility.

Most recently, Löffler et al. [2,3] trained a Siamese Network for fast similarity-based scene retrieval. Their learned embedding is approximately distance-preserving at a much lower dimensionality than the original unstructured ensemble of trajectories. Their Siamese Network is a Temporal Convolutional Network with a gating structure, and their study explores different heuristics for channel assignments [2]. Their later work proposes a dynamic ellipsoid channel assignment method that outperforms the previous assignment heuristics [3]. The authors focus only on professional soccer tracking data. For this sport, their similarity search enables retrieval times several orders of magnitude lower than the previous state-of-the-art method, Chalkboarding [1].

### 2.2. Effective Sample Mining

Metric learning depends on effective sample mining, as the choice of instances influences convergence and generalization. An important line of investigation is triplet mining, with concepts extending to pairwise learning, such as the contrastive loss used in this work.

A key concept in sample mining is sample difficulty. Based on the idea of pair or triplet difficulty, i.e., easy or hard positives or negatives, Xuan et al. [22] highlight the complementary role of easy positives. Such samples help form compact clusters but avoid over-clustering, in contrast to mining of only the hardest positives or negatives [23]. Schroff et al. [14] popularized the use of semi-hard negatives. These instances are farther from an anchor than positive instances, but within a margin. A recent proposal was adaptive sampling according to a curriculum [24], where training begins with easy triplets and progresses to harder ones later. We adapt semihard sampling to serve as a baseline for pairwise similarity learning.

### 2.3. Active Learning

Active Learning reduces annotation cost for unlabeled pool datasets by selecting only the most informative samples for labeling by an oracle [10]. It operates in a loop that starts with an unlabelled sample pool, a model that learns a task, a sample selection method, and a (human) oracle or annotator that annotates samples and adds them to the model’s training dataset. These iterative algorithms are usually guided by the model’s signals, which sample active selection heuristics considered relevant for learning. AL complements triplet or pair mining, as discussed in Section 2.2.

In this paper, we review the sampling methods from the three principal families applicable to Deep Learning: uncertainty-based, diversity-based, and hybrid approaches that combine the two [25]. As a baseline, Random Sampling selects data uniformly at random from the unlabeled pool. Representative of the uncertainty-based methods, Gal et al. [26] propose MC Dropout, which estimates a neural network’s predictive uncertainty by performing multiple stochastic forward passes with dropout enabled, effectively sampling well-calibrated uncertainty for unlabeled samples. The related method Entropy sampling [10] selects samples with the highest entropy in the predictive class distribution, corresponding to the points of greatest uncertainty. The mining of effective samples for classification and object detection on image data has uncertainty-based sampling to exclude non-informative training samples [27]. The authors used ensembles of hundreds of models to estimate the uncertainty of each instance in a dataset and excluded samples with high certainty from training. Other works predict per-instance Dynamic Uncertainty scores [28]. However, learning to predict such scores requires full training. Hence, more recent works extrapolate these scores from a few instances [29]. For diversity-based methods, CoreSet [17] selects a batch of samples that covers the set of the batch by leveraging embeddings from the last dense layer before the network’s output. Hence, they address the *K*-Center problem to select samples that best cover the representation space. For the hybrid family, we select BADGE [11], which computes gradient embeddings with respect to pseudo-labels and then applies k-means++ clustering, selecting instances with diverse gradients and high magnitudes, thereby encouraging both sample diversity and high uncertainty. This sampler addresses the pathological consequences of relying only on uncertainty-sampling [11,30,31], which tends to sample batches of nearly identical samples. Instead, the authors promote gradient diversity, which induces large and diverse changes in the model. We adapt BADGE to the pairwise sampling task and include the other sampling techniques in our experimental study.

## 3. Background

This section first provides an example of trajectory similarity search. Then, it defines a general notation for the trajectory data that this study experiments with. Next, it reviews the assignment problem and the costly optimization provided by the Hungarian algorithm [32]. Finally, the section outlines how the learning task of the Siamese Network includes the Hungarian algorithm’s solution, i.e., the assignment.

**Figure 1:**
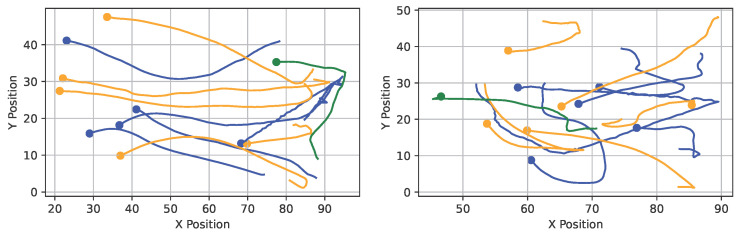
The two basketball scenes show trajectories of 5 players per team (blue and yellow trajectories) and a basketball (green trajectory). To calculate their similarity involves two steps [2]: (i) the two scenes’ trajectories are assigned optimally using the Hungarian algorithm [32], and (ii) their pairwise Euclidean distances are summed up.

Trajectory similarity search aims to retrieve the top-N most similar scenes with respect to a distance metric [2]. Given the two exemplary basketball scenes in Figure 1, one represents a query scene. The other represents a sample from a large dataset; the similarity requires two steps: first, the optimal assignment of trajectories between scenes is calculated via the Hungarian algorithm [32], and second, the sum of their (Euclidean) distances is produced. This step repeats for all scenes in the dataset, and the top N results are returned.

We formally define multi-trajectory sport scenes as follows. For comparability, we adapt the notation proposed by Löffler et al. [2] to our data. We represent each trajectory as a spatio-temporal matrix $x\in R^{S\times T}$:

$$x=\left[\begin{array}{cccc} x_{1,1} & x_{1,2} & \ldots & x_{1,T}\\ x_{2,1} & x_{2,2} & \ldots & x_{2,T}\\ \vdots & \vdots & \ddots & \vdots \\ x_{S,1} & x_{S,2} & \ldots & x_{S,T} \end{array}\right],$$

where *S* denotes the spatial dimension of the trajectory (S=2 for 2D positional tracking), and *T* the temporal length in frames. For example, we set T=150 for 6 s of tracked data at 25 Hz in basketball. Each row $x_{s,1:T}$ corresponds to one spatial coordinate (e.g., *x* or *y*) over time. A scene with multiple agents is denoted by $X\in R^{N\times S\times T}$, where *N* is the number of tracked entities. Thus, for an American football scene with 10 players plus the ball, X has shape 11×2×150. For an American football play with 22 players plus the ball, the representation is 23×2×50, i.e., tracking at 10 Hz for 5 s scenes.

To calculate distances between pairs $X_{1}$ and $X_{2}$ of scenes, many sports do not define a fixed assignment of the two matrices’ trajectories $x_{n}$ (n∈N) from $X_{1}$ and $x_{m}$ (m∈N) due to the high dynamics in many team sports [2,7,33]. Despite the existence of roles in basketball, the game is also highly dynamic. In American football, the roles are generally more rigid for specific groups. To retain the generalizability of our method, we do not assume fixed assignments.

To calculate the similarity between two scenes, Löffler et al. [2] use the Hungarian algorithm to calculate row-wise assignments in dimension *N* of both scenes, yielding a ground-truth distance. The authors use the Euclidean distance as a metric and sum up all distances for pairs of matrices after assignment.

(1) $$d\left(\vec{x},\vec{x^{\prime }}\right)=\frac{1}{T}\sum_{t=1}^{T}\left|\right|{\vec{x}}_{:,t}-{\vec{x^{\prime }}}_{:,t}{\left|\right|}_{2}$$

This ground-truth computation is an important cost driver of training Siamese Networks on these combinatorial datasets: (1) pairwise distances have the complexity of $O(N^{2}\;\cdot \;S\;\cdot \;T)$ where S×T is matrix size, (2) and the Hungarian algorithm has the complexity of $O(N^{3})$. Hence, the overall complexity is $O(N^{2}\;\cdot \;S\;\cdot \;T+N^{3})$. Faster implementations, such as Jonker–Volgenant (JV) [34] or the heuristic Auction algorithm [35], can also be used in conjunction with our proposed method. While JV has the same asymptotic complexity, $O(N^{3})$, it is more efficient in practice, and the Auctioning Auction algorithm provides a fast, approximate solution. We leave these technical optimizations for future work.

Following Löffler et al. [2,7], we compute the ground-truth assignments and distances optimally for any pairwise comparison. The Siamese Network’s inputs, however, do not use any special heuristic to assign the matrices’ rows to the neural network’s input channels. In contrast to previous work, we do not explore role-/grid-based [2] or data-driven [7] assignments. Instead, we employ random channel assignments that maximize generalizability across diverse sports and do not introduce any bias that may compromise the validity of our experiments. Hence, we do not use stable ordering, as is common in color channels of image data, but instead use a random ordering that varies between games.

Table 1 consolidates all symbols and variable definitions used within this paper.

## 4. Method

Training a Siamese Network using a full dataset of all possible pairs of sport scenes is prohibitively expensive due to the combinatorial complexity of pairwise comparisons and the associated cost of computing the Hungarian algorithm’s optimal distance for high-dimensional trajectory pairs. For example, given *N* = 100,000 scenes, a full training would train on $\frac{N\;\cdot \;(N-1)}{2}$ or approximately 5 billion pairs. Due to this cost, our experiments instead rely on “simulated full training” by generating large amounts of pairs, e.g., 10 million pairs for *N* = 100,000 scenes, i.e., 0.2% of all possible combinations. Simulated full training, thus, is a practical and very large upper bound, whereas the true combinatorial set is intractable.

In practice, random subsampling of pairwise comparisons is the standard method for training models until convergence. However, training with data generated by random sampling can be inefficient due to data redundancy and low information content [10]. While the cost compared to full training diminishes, the learned representation may be of lower quality for harder, rarer, or more uncertain samples.

In this work, we propose using more advanced sampling methods for sample mining by transferring Active Learning samplers that promise to select more informative samples, thereby improving the retrieval quality of sport scene embeddings and delivering higher-quality search results at lower cost.

Conceptually, we extend sample mining by transferring the Active Learning loop to pairwise representation learning. For this work, we start with a pool of scenes (not pairs of scenes) and sample informative pairs for which we still need to compute the distances. This pool of scenes is similar to the unlabelled pool. Instead of an oracle, we have the costly Hungarian algorithm to assign a cost to a pair of samples. Analogously to Active Learning, we propose selecting informative pairs using the model and a sampling method, thereby reducing distance computation and approaching the oracle’s cost.

**Figure 2:**
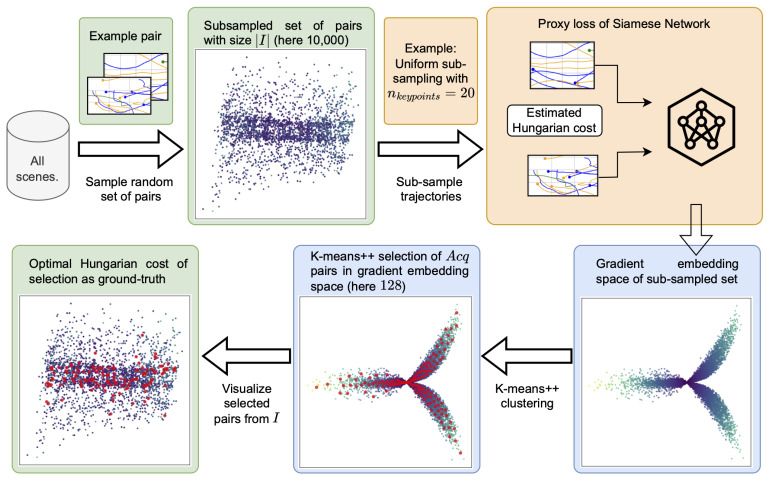
This figure illustrates how our method selects informative pairs. First, a large set of randomly sampled pairs of size |I|=10,000 is subsampled from all possible pairs across all individual scenes, shown here in 2D after Principal Component Analysis (PCA). Second, the scenes are efficiently subsampled using $n_{keypoints}$ uniformly distributed keypoints. Third, the estimated Hungarian cost between pairs is the hallucinated label to obtain a proxy loss for the Siamese Network. Based on this loss, we construct the gradient embedding space, here shown in 2D after PCA (heatmap encodes magnitudes). This space encodes information on the model’s uncertainty (via the gradient vector magnitudes) and sample diversity (via the gradient vector directions) [11]. The selection itself relies on K-means++ to quickly obtain Acq=128 pairs. Finally, the Siamese Network trains using the ground-truth Hungarian cost for these pairs. The boxes in green signify the original Euclidean trajectory space and blue the embedding space. Orange signifies the Siamese Network’s encoding step.

Figure 2 illustrates our proposed method as a simple step-by-step flowchart. Our method initially samples a large set *I* of pairs from the pool. Then, the active sampler calculates which of these pairs are most suitable for learning. Our method uses gradient embeddings of pairs, generated from cheap-to-compute proxy losses, to select Acq=128 diverse and uncertain samples. For these 128 pairs, the true Hungarian cost serves as ground truth for training. The remainder of this chapter formalizes our approach.

For Active Learning samplers, in the AL loop, the methods typically evaluate the “quality” of each sample from the unlabeled pool. In our context, this is not possible due to the combinatorial nature of pairwise comparisons. Sub-sampling of the full dataset is mandatory. From the smaller subset of possible pairs, the samplers then select the most informative instances for “labeling” by the Hungarian algorithm.

In our problem definition, one iteration of the random-sampling epoch executes as follows: The sampler first obtains a subset of scenes from the pool and selects Acq random pairs for annotation. Next, the pairwise distances for these Acq pairs are computed via the Hungarian algorithm, and the model trains on the pairs with associated distances. At the end of the epoch, all pairs of scenes from the pool have been evaluated, and the epoch concludes. It is important to note that the model training cannot consider all possible pairs of scenes. In the following, we describe alternative methods selecting pairs of scenes for training actively.

(1) Uncertainty-based sampling: The traditional uncertainty-based samplers for Deep Neural Networks are designed for selecting samples based on the entropy of class probability distribution, e.g., via a Softmax activation function, such as Entropy sampling [10]. We adapt this estimation of the predictive uncertainty of the Siamese Network for a pair of samples by simulating Bayesian uncertainty through Monte Carlo (MC) Dropout [26].

Given an input of scenes $X_{1}$ and $X_{2}$, and their embeddings $f(X_{1})$ and $f(X_{2})$, we apply random Dropout masks at *P* forward passes during inference to generate stochastic predictions $\mathrm{preds}=d\left(f{\left(X_{1}\right)}^{p},f{\left(X_{2}\right)}^{p}\right))\;\forall p\in \left\{1,\ldots ,P\right\}$. The distribution of the predictions then exhibits the variance

(2) $$\sigma^{2}=\frac{1}{P}\sum_{p=1}^{P}{\left({\mathrm{preds}}^{\left(p\right)}-\mathrm{mean}\left(\mathrm{preds}\right)\right)}^{2}.$$

Given a set of pairs *I* from the pool’s subset, we can rank these by their predictive variance $\sigma_{i}^{2},i\in \left\{1,\ldots ,I\right\}$ to select the Acq samples with the highest variance.

We can also apply Entropy sampling by computing the entropy of a pair

(3) $$H=\frac{1}{2}\log\left(2\pi e\;\cdot \;\sigma^{2}\right)$$

assuming the distribution to be Gaussian, and selecting Acq of the subset *I*’s pairs with the highest entropy. These methods were shown to select uncertain samples most efficiently when the selection size Acq was smaller, but produced redundant selections or focus on rare instances, such as outliers, when the selection size Acq was larger [25,31].

(2) Representative Sampling: The second type of method, which we adapt to the problem of pairwise representation learning, is the CoreSet sampler [17]. In an initial step, we predict the embeddings of the pairs of scenes $\left(X_{1},X_{2}\right)$ in the subset *I* by applying the model *f* as $e_{1}=f\left(X_{1}\right)$ and $e_{2}=f\left(X_{2}\right)$, and concatenating the embeddings $\left(e_{1},e_{2}\right)$, yielding a vector per pair. Next, the step to achieve a representative sampling of these embedding vectors is outlined. Sener and Savarese [17] apply *K*-Center greedy clustering with Euclidean distance to find Acq samples from the subset *I* that are most representative of the data.

This sampling method was shown to effectively select sets of Acq samples that are representative of the dataset distribution. However, the selected instances may have low information value and be redundant compared to the already annotated training data, leading to slower convergence [25,30,31].

(3) Pairwise Diverse and Uncertain Gradient (PairDUG): To resolve the issues of uncertainty-based and diversity-based sampling, i.e., the focus on outliers or instances of low information value, several methods seek to combine the concepts [11,30]. In this work, we adapt the gradient-based BADGE [11] to the novel learning task or pairwise metric learning. BADGE leverages gradient signals to select Acq samples from a subset *I*. It selects uncertain samples, i.e., those with a high gradient magnitude, and those that are diverse, i.e., with gradients in the embedding space that are spread out. BADGE is designed for classification and uses the model’s own predictions to generate proxy labels used for loss calculations. We extend the sampler to pairwise comparisons.

To compute gradients, we would actually require ground-truth distances for pairs $X_{1}$ and $X_{2}$. However, that would require solving the assignment problem using the Hungarian algorithm, with complexity $O(I^{3})$, which would be practically intractable. We dub this costly variant “PairDUG gt” (gt for ground truth) and use it in our experiments as a performance benchmark for the PairDUG concept.

Instead, we simplify the distance and solve it near-optimally, resulting in “PairDUG fast”. For each pair $\left(X_{1},X_{2}\right)$ we uniformly subsample $n_{\mathrm{keypoints}}$ time indices $\left(t_{1},\ldots ,t_{n_{\mathrm{keypoints}}}\right)$, resulting in smaller $\left(X_{1}^{\prime },X_{2}^{\prime }\right)$. In a pre-study, we compare the optimal number of keypoints on a subset of pairs for which we calculate ground-truth distances, and optimize $n_{\mathrm{keypoints}}$ to achieve low Mean Absolute Error (MAE) and low Mean Absolute Percentage Error (MAPE) at a low computational cost.

Using this approximation, the cost matrix between trajectories *j* and *k* of $X_{1}^{\prime }$ and $X_{2}^{\prime }$ with length *T* is computed as

(4) $$C_{jk}=\frac{1}{T}\sum_{t=1}^{T}{∥X_{1}^{\prime }\left(t,j\right)-X_{2}^{\prime }\left(t,k\right)∥}_{2}.$$

Then, the Hungarian algorithm finds the minimal assignment that acts as the pairwise distance as

(5) $${\mathrm{cost}}_{fast}=\min_{\pi }\sum_{j}C_{j,\pi (j)}.$$

Next, we compute the loss of the Siamese Network *f* under training as follows. We calculate the Euclidean distance *d* of the embeddings $e_{1}$ and $e_{2}$ of the pair $X_{1}$ and $X_{2}$ and use the proxy distance ${\mathrm{cost}}_{fast}$ as a stand-in label in place of the real ground-truth cost to compute the gradient approximation of the instance’s loss δ as

(6) $$\delta =2\times (d\left(e_{1},e_{2}\right)-{\mathrm{cost}}_{fast}).$$

This function is the derivative of the squared error loss. Finally, we calculate the gradient embedding following [11] by taking the derivative of the loss with respect to the parameters $\theta_{emb}$ of the final (or embedding) layer

(7) $$\mathrm{grads}=\frac{\nabla L}{\nabla \theta_{emb}}\;\cdot \;L\left(f,{\mathrm{cost}}_{fast}\right).$$

The resulting gradient embedding vector compactly represents the magnitude and direction of the gradients. It has beneficial properties that we can exploit to find diverse data that the model is uncertain about, i.e., has a high gradient magnitude in the embedding layer. On the gradient embedding, we apply K-means++ clustering to determine Acq representative centroids and to select the nearest sample for each centroid [11]. Relying solely on gradient magnitude would result in selections that are vulnerable to gradient fluctuations, common in real applications with noise. The selection of diverse gradient vectors mitigates this instability and maintains robust convergence in learning. These Acq pairs are the selection of the PairDUG method.

To elaborate on the error propagation between PairDUG fast and gt, we first determine the error as $\varepsilon ={\mathrm{cost}}_{fast}-{\mathrm{cost}}_{gt}$, where ${\mathrm{cost}}_{gt}$ is the optimal Hungarian cost without sub-sampling and ${\mathrm{cost}}_{fast}$ is the estimation with sub-sampling. During training, the gradient magnitude used by PairDUG is calculated via Equation (6), from which follows that the approximation error of δ is bounded by $\delta_{fast}-\delta_{gt}=-2\varepsilon$, where ε is proportional to the distance approximation error. Furthermore, assuming that ε is approximately zero mean and its magnitude decays with growing $n_{keypoints}$, ε does not distort the selection that PairDUG fast performs. In addition, K-means++ clustering is invariant to uniform translations in gradient space, and only relative magnitude errors affect PairDUG fast’s selections.

We quantify the empirical distribution of ε for Uniform sub-sampling in Section 7.4.1, and find a Mean Absolute Percentage Error of 0.01 and a correlation of 0.9997 for basketball and, respectively, 0.0027 and 0.99997 for American football (using 1000 random trajectory pairs and n=20 keypoints). Consequently, the gradient’s bias is negligible compared with natural gradient variance within a batch; see Section 7.2 for an analysis of gradients’ variance within a batch for both variants PairDUG fast and gt.

The computational complexity of the proposed method is discussed in the Appendix A.

## 5. Datasets

The two datasets we used are the raw SportVU NBA Game Logs from the 2015–2016 games [13] (Available at https://github.com/linouk23/NBA-Player-Movements, accessed on 10 November 2025), featuring basketball tracking data, and the NFL’s Next Gen Stats tracking data from the NFL 2018–2019 seasons, that is formatted like the Big Data Bowl (https://operations.nfl.com/gameday/analytics/big-data-bowl/, accessed on 10 November 2025) from 2019 [12], featuring highlights (available at https://github.com/asonty/ngs_highlights, accessed on 10 November 2025) of American football. We had to exclude soccer due to the proprietary nature of sufficiently large datasets [2,3].

### 5.1. Basketball Dataset

Basketball was selected as the first sport for evaluation due to its large amount of available tracking data. Each game involves ten players (five per team) and the ball, for a total of eleven tracked entities. The trajectory data can therefore be represented as a matrix of size 11×T, where *T* denotes the number of temporal positions recorded for each entity.

**Figure 3:**
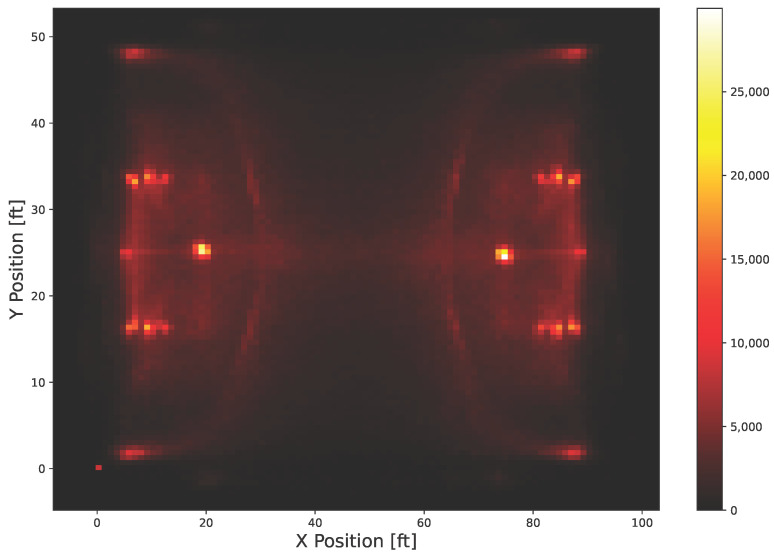
This figure visualizes the basketball trajectory dataset’s players’ and ball positions as a discrete location heatmap using 10,000 randomly sampled scenes X. Several strategic locations on the basketball court appear more frequently, such as the free-throw line located 15 feet (4.57 m) from the backboard.

For our experiments, we used tracking data from the NBA 2015 and 2016 seasons, comprising moments from 631 games. The data contains metadata and event-level information. The trajectories themselves are visualized in the heatmap in Figure 3 and were recorded using six cameras. The relevant attributes for trajectory reconstruction are the “eventId” (the scene identifier) and “moments” (a time series of entity positions). Positions are given in feet and were recorded at 25 frames per second.

Since event durations vary, scenes were segmented into a fixed window of six seconds (150 frames). Each scene was represented by the matrix X of the shape (11×2×150) for S=2 dimensional tracking data.

### 5.2. American Football

American football was selected as the second sport due to its structured formations and a larger number of entities than basketball. According to the regulations, a single play involves 22 players plus the ball, for a total of 23 tracked entities. The trajectory data can therefore be represented as a matrix of size 23×2×T, where *T* denotes the temporal positions of each entity during the play, which is set to T=50 for 5 s long segments. Player positions are measured in yards.

**Figure 4:**
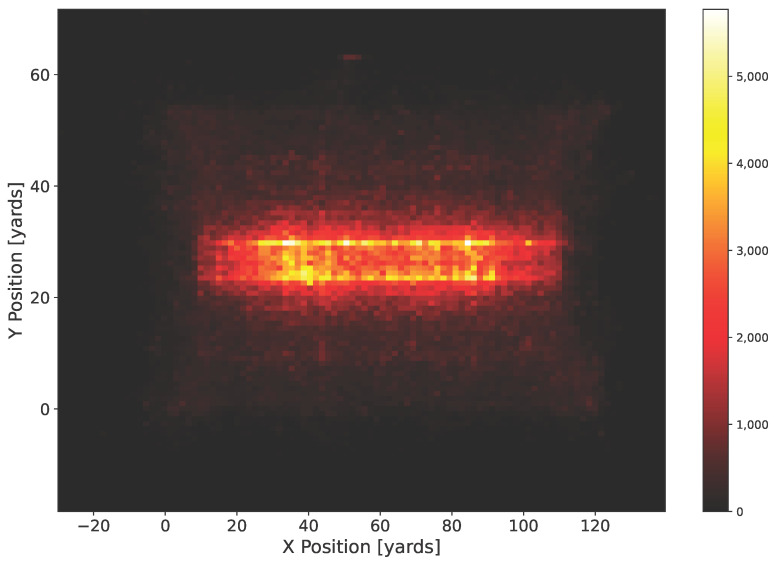
This figure visualizes the American football trajectory dataset’s players’ and ball positions as discrete heatmaps using 10,000 randomly sampled scenes X. The heatmap shows the defensive and offensive lines for each team across the width of the field, with players lining up near the horizontal “hash marks,” and the narrow so-called “line of scrimmage” as a small vertical gap between the lines.

The data were collected from the NFL 2018–2019 season via the Next Gen Stats (NGS) tracking system, an RFID-based positioning system with a 10 Hz sampling rate, and comprised 561 highlight plays. The plays themselves are visualized in the heatmap in Figure 4.

### 5.3. Pair Generation

For our experiments, we split the recorded games into train, validation, and test sets; see Table 2. For basketball, we use the data from 510 games for training, 63 for validation, and 63 for testing. The data is partitioned to ensure independence across teams and games. This amounts to 99,327, 12,360 and 12,366 scenes of 6 s each. Then, instead of using all combinations, which would be computationally infeasible due to the combinatorial growth, we construct large subsets. To obtain fixed datasets for fair comparisons, we generated scene pairs and computed their ground-truth distances with the Hungarian algorithm. Specifically, we randomly sample 10 million pairs of basketball scenes for training from the 510 games, and 500,000 each for validation and testing from their games.

For American football, we split the data along games as well. The total sum of 561 plays is separated into 449 for training, 56 for validation, and 56 for testing. This amounts to 3106, 384 and 386 scenes of 5 s each, with no set’s games overlapping. Finally, we randomly sample 100,000 pairs of American football scenes for training, validation, and testing.

We decided to draw the validation and test samples from games excluded from the training dataset. We consider this a more representative evaluation method than sampling from different scenes within the same games, because it avoids information leakage from similar player positions.

## 6. Experimental Design

### 6.1. Configuration

For all experiments, we use PyTorch (version 2.9.0) [36] together with the Adam optimizer [37], with an initial learning rate of $\eta =1\times 10^{-3}$ and weight decay $\lambda =1\times 10^{-5}$. Models are trained for 500 epochs with early stopping based on validation loss and 10-epoch patience, subsampling the training data to 10,000 samples for basketball and 1000 for American football. We refer to Section 7.4 for an evaluation of different subset sizes. Then we actively select (“acquire” in Active Learning) the most informative set of samples with a fixed size of Acq=128 throughout all our experiments. We employ a Siamese Network with three fully connected layers (input →256→128→64) and ReLU activations, with optional dropout layers (with the probability of p=0.3) depending on the chosen active sampling strategy. All experiments were performed on AMD Ryzen 9 7950X3D 16-Core processors and NVIDIA GeForce RTX 4070 Ti Super GPUs with 16 GB of VRAM.

We use a standard loss function based on the Euclidean distance with two regularization terms [2]. The first term penalizes large predictions for the two scenes, $X_{1}$ and $X_{2}$, and the second penalizes large weights, θ, both using the $l_{2}$ norm

(8) $$L\left(X_{1},X_{2}\right)=\left(|\right|f_{\theta }\left(X_{1}\right)-f_{\theta }\left(X_{2}\right){\left|\right|}_{2}-d\left(X_{1},X_{2}\right){)}^{2},$$

i.e., the MSE, and including two regularization terms:

(9) $$\left|\right|f_{\theta }\left(X_{1}\right){\left|\right|}_{2}+\left|\right|f_{\theta }\left(X_{2}\right){\left|\right|}_{2}+{\left||\theta |\right|}_{2}.$$

As is common for Siamese Networks, we use identical weights for each branch of the network’s architecture to predict each scene’s embedded representation $f_{\theta }\left(X\right)$, on which we apply the loss function in direct comparison with the known ground-truth distances [38], learning a distance-preserving embedding [2].

### 6.2. Evaluation Metrics

To validate the optimized training strategy’s performance, we evaluate the retrieval quality of the more efficient training loop; see Section 6.2.1. Next, we inspect the gradient signals to show and interpret the underlying differences in the Siamese Network’s gradients that enable the efficiency gains; see Section 6.2.2.

#### 6.2.1. Retrieval Quality Metrics

Retrieval quality metrics must assess the quality of the learned lower-dimensional representation relative to the original dataset. There are several critical dimensions to consider for the application of sports scene retrieval. Previous work [2] considers the structural correspondence as well as the ordering and neighborhood structure as essential characteristics of the representations.

(1) Mean Absolute Percentage Error (MAPE): The structural correspondence measures the total distance error between pairs of scenes, comparing the ground-truth distance of scenes $X_{1}$ and $X_{2}$ with the distance of the embedded scenes $f(X_{1})$ and $f(X_{2})$.

(10) $${\mathrm{MAPE}}_{D}=\frac{1}{\left|D\right|}\sum_{i,j}^{\left|D\right|}\left|\frac{d\left(X_{i},X_{j}\right)-d\left(f\left(X_{i}\right),f\left(X_{j}\right)\right)}{d(X_{i},X_{j})}\right|.$$

(2) Top-N Mean Spearman Correlation Coefficient (MSRCC): The ordering and ranking of retrievals is an important property when it comes to search. We estimate ranking quality by randomly sampling 1000 query instances from the test sets and calculating the ground-truth distances to 200 randomly selected instances for each query. A full computation of the test sets would be prohibitively expensive. Then we calculate the top-N Mean Spearman Correlation Coefficient to evaluate the scene ordering between the ground-truth and the learned embedding as follows. For one of the 1000 query scenes, we select *N* other instances $\left\{X_{0},\ldots ,X_{N}\right\}$ and calculate their ground-truth distances to the query $E=\{d\left(X_{query},X_{0}\right),\ldots ,d\left(X_{query},X_{N}\right)\}$. Next, we compute their distance in the learned embeddings $\hat{E}=\{d\left(f\left(X_{query}\right),f\left(X_{0}\right)\right),$ …$,d(f\left(X_{query}\right),f\left(X_{N}\right))\}$. With these two distance lists, we can compute their Spearman rank correlation coefficient $r=r(E,\hat{E})$ and then average these for all 1000 queries to obtain the MSRCC for N=1000 other instances from the test dataset.

#### 6.2.2. Gradient Quality Metrics

We evaluate the quality of a batch of samples by their gradients during model training. This provides insights into possible reasons for differences in the performance of the sample selection method. We measure the quality of gradients primarily along two dimensions: diversity and magnitude. In addition, we closely examine their coefficient of variation and Signal-to-Noise (SNR) ratio for a more detailed analysis.

(1) Mean Gradient Norms: First, we calculate the instance’s mean loss as follows. Let $g_{i}\in R^{F}$ denote the flattened gradient of the loss with respect to model parameters for the *i*-th example in a set of size Acq. We then define it as the arithmetic mean of the per-instance gradient vectors.

(11) $${∥g_{i}∥}_{2}=\sqrt{\sum_{f=1}^{F}g_{i,f}^{2}}.$$

This vector represents the overall direction and magnitude of the parameter update. If many per-instance gradients cancel out, the mean will be small even if individual gradients are large. Thus, the mean gradient norm ${\bar{∥g∥}}_{2}$ is the arithmetic mean of the $l_{2}$-norms of the per-instance gradients [39] as

(12) $${\bar{∥g∥}}_{2}=\frac{1}{K}\sum_{i=1}^{K}{∥g_{i}∥}_{2}.$$

This metric measures the typical magnitude of a training batch; small values suggest vanishing gradients, while large values suggest strong updates.

(2) Gradient Diversity: Gradient Diversity [40] is the average pairwise cosine distance between per-instance gradients. This metric captures directional disagreement across instances in a batch. High values indicate that gradients point in diverse directions, whereas low values indicate that they align more closely. We first normalize per-example gradients

(13) $${\tilde{g}}_{i}=\frac{g_{i}}{∥g_{i}{∥}_{2}},$$

then compute Gradient Diversity ${\bar{D}}_{g}$ as follows:

(14) $${\bar{D}}_{g}=\frac{2}{N(N-1)}\sum_{1\leq i<j\leq N}\left(1-{\tilde{g}}_{i}^{⊤}{\tilde{g}}_{j}\right)$$

(3) Coefficient of Variation: This metric is defined as the ratio of the standard deviation of gradient norms to their mean. We use it to quantify the relative variability in the gradient magnitudes across instances in a batch. Here, high values indicate that the gradients differ greatly in magnitude across instances. We define it as

(15) $${\mathrm{CV}}_{g}\;=\;\frac{\sqrt{\frac{1}{N}\sum_{i=1}^{N}{\left(∥g_{i}{∥}_{2}-{\bar{∥g∥}}_{2}\right)}^{2}}}{{\bar{∥g∥}}_{2}+\varepsilon }$$

where $\varepsilon \approx 10^{-8}$ is a small constant for numerical stability.

(4) Gradient Signal-To-Noise Ratio: This ratio is between the mean gradient norm (i.e., the signal) and its standard deviation (i.e., the noise). A higher SNR means individual gradients are more consistent in magnitude, while a low SNR suggests that the batches’ gradients are highly variable [41]. We define it as

(16) $${\mathrm{SNR}}_{g}\;=\;\frac{{\bar{∥g∥}}_{2}}{\sigma_{g}+\varepsilon }$$

## 7. Experiments

We first present the results on retrieval quality in Section 7.1, beginning with the larger basketball dataset, and then showing a more detailed analysis for the American football retrieval quality. Next, Section 7.2 sheds light on the behavior of the samplers and gradient signals. The proof of lower training duration is provided in Section 7.3 and a thorough ablation study is given in Section 7.4, where the hyperparameters and components of the proposed sampler are analyzed.

### 7.1. Retrieval Quality Results

We present the experimental results for the retrieval quality of basketball in Section 7.1.1 and of football in Section 7.1.2.

#### 7.1.1. Basketball Retrieval Quality

The overall idea of this experiment is to demonstrate that our active sampling method, PairDUG fast, performs better than other samplers, including random sampling. Additionally, we show that the fast PairDUG variant with approximated distances is on par with the more costly ground truth (gt) version.

For the analysis, we train a Siamese Network on the basketball dataset of 10 million pairs and use a subset *I* of size 10,000, from which the samplers select Acq=128 instances for training. For these pairs, the Hungarian algorithm delivers the ground-truth distance. After training, we evaluate the retrieval quality on a test set of 500,000 samples using the MAPE metric to assess overall structural correspondence and the MSCRR metric to demonstrate retrieval quality.

**Figure 5:**
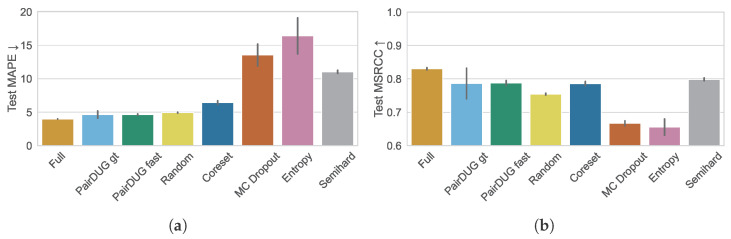
Error metrics MAPE and MSCRR for the test set of the basketball dataset. Full training is the simulated upper-bound baseline that was trained on all 10 M pairs, and random is the sampling method to beat, which sub-sampled the 10 M samples. (**a**) shows that both PairDUG variants accomplish competitive performance with respect to the baselines “simulated full training” and random, while other samplers fail on the MAPE metric. Only Semihard and Coreset achieve competitive performance in the MSCRR metric in (**b**), i.e., in the evaluation of the ordering of retrievals.

Figure 5a shows the results for the metric MAPE for each method, averaged over five repetitions. The “simulated full training” achieved a test MAPE of 3.98±0.01 after training on 10 million pairs. We consider random sampling as the baseline method that other samplers must beat. This sampler picks batches of 128 pairs at random and the Siamese Network trains on a total of 1,280,000 samples, or 12.8% of the original training dataset. Random achieved a MAPE of 4.93±0.07. The “PairDUG gt” method, which is trained with optimal but costly distances, outperforms full training with a MAPE of 4.64±0.55. Our proposed method “PairDUG fast” achieves a slightly lower MAPE of 4.63±0.11 with fast distance approximation, still performing better than random subsampling. We explain our methods’ excellent performance due to the reduction of noise and the focus on difficult and representative pairs, whereas random training is negatively affected by lower-quality training data. Other samplers fail to beat random sampling. Coreset only obtains 6.43±0.28, and the samplers MC Dropout (13.53±1.64), Entropy (16.40±2.70), and Semihard (11.04±0.24) fail to achieve competitive scores.

Figure 5b shows the results for the metric MSCRR for each method, averaged over five repetitions. “Full” represents the upper bound of 83.03±0.33, and is calculated for 1000 different query samples and 200 gallery samples. Random sampling has a lower score of 75.43±0.29 but is more stable. The two PairDUG variants, gt (78.62±4.58) and fast (78.76±0.74), again obtain highly competitive ordering results, with Coreset (78.58±0.64) following. Interestingly, Semihard sampling is competitive in the ordering, with MSCRR of 79.85±0.43, but fails to capture the overall structure, resulting in a high MAPE. The uncertainty-based samplers MC Dropout (66.70±0.74) and Entropy (65.57±2.44) trail the field.

**Figure 6:**
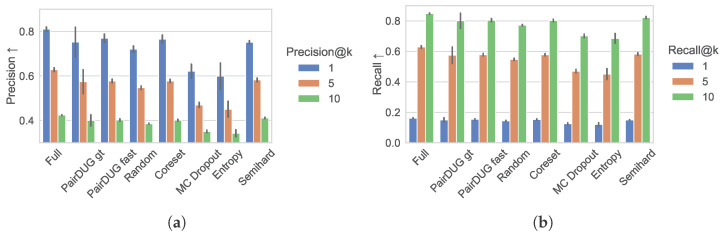
The Precision@*k* metric for k∈(1,5,10) in (**a**) and Recall@*k* metric for k∈(1,5,10) in (**b**) for the test set of the basketball data show the retrieval quality of the top-k search results compared with the ground-truth retrievals using optimal Hungarian distances.

Figure 6a,b show the results for the Precision@*k* and Recall@*k* metrics for the values k∈(1,5,10). The Siamese network trained on the simulated full dataset achieves a Precision@1 of around 0.8, which diminishes for k=5 and k=10. Both PairDUG variants, as well as Coreset and Semihard sampling, achieve results comparable to PairDUG’s, though slightly worse, and beat Random sampling. The Recall@*k* results confirm this grouping of methods.

These results show that only the combination of diverse and uncertain sampling in PairDUG consistently outperforms random subsampling and is at least on par with simulated full training. We infer a positive contribution of diversity-based sampling from the performance of Coreset, while uncertainty-based samplers fail.

#### 7.1.2. Football Retrieval Quality

The second experiment demonstrates that our active sampling method, PairDUG (gt and fast), also transfers to other datasets from the sports search domain, showing its generalizability to other complex trajectory datasets.

In our analysis, we train a Siamese Network on the American football dataset of 100,000 pairs and use a subset *I* of size 1000, from which the samplers select Acq=128 instances for training. We refer to Section 7.4 for ablation studies on the size of *I*. Then, we evaluate retrieval quality on a test set of 100,000 samples.

**Figure 7:**
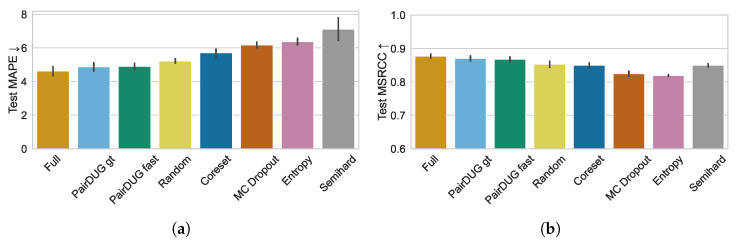
Error metrics MAPE and MSRCC for the test set of the American football dataset. We present the two baseline methods “full training” of a model on all 100 k pairs and “Random”, a model trained on a randomly sub-sampled set of 12.8 k pairs. The evaluation on the MAPE metric in (**a**) demonstrates that both PairDUG variants accomplish competitive performance with the same small budget of 12.8 k pairs, while other samplers fall behind. Similarly to basketball data, (**b**) shows that only Semihard and Coreset achieved competitive MSRCC scores on the American football data.

Figure 7a shows the results for the metric MAPE for the samplers, averaged over five repetitions. The full training serves as a baseline and achieved a test MAPE of 4.62±0.27. Random achieved a MAPE of 5.23±0.13, which the other samplers have to beat. Our proposed PairDUG variants achieve a competitive MAPE of 4.88±0.25 for gt and 4.90±0.18 for fast, with only Coreset performing in the range of Random sampling with its MAPE of 5.71±0.22. Other samplers fail to beat Random sampling. Figure 7b mirrors the results with our method PairDUG, leading the sampling methods with regard to MSCRR scores.

**Figure 8:**
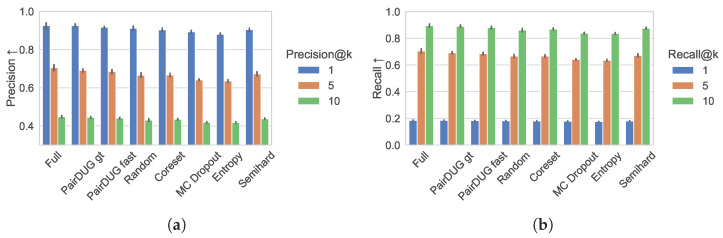
The Precision@*k* metric for k∈(1,5,10) in (**a**) and the Recall@*k* metric for k∈(1,5,10) in (**b**) for the test set of the American football data show the retrieval quality of the top-k search results compared with the ground-truth retrievals using optimal Hungarian distances.

Figure 8a,b show the results for Precision@*k* and Recall@*k*. With the simulated full training, the Siamese Network’s embeddings encode sufficient information to achieve over 0.9 in top-1 precision. The PairDUG variants follow behind, with Precision@5 having larger margins over stronger baselines, such as Random and Semihard sampling. The recall performance mirrors the results on precision.

This evaluation on the second dataset from the sports domain confirms the viability of PairDUG as a suitable sampling method for cost reduction in sports scene retrieval.

#### 7.1.3. Statistical Significance Testing

This analysis assesses whether the performance differences between each sampling strategy and the baseline Random selection are statistically significant, ensuring that observed improvements are not due to random variation in the data splits.

For the evaluation metrics MAPE and MSRCC, we perform independent two-sample *t*-tests comparing the distribution of results for every method against the Random baseline. The test is Welch’s *t*-test, which does not assume equal variances between groups.

The resulting *t*-statistics and *p*-values quantify the direction and strength of each difference. A negative *t* in the metric MAPE indicates lower error relative to the baseline, and a positive *t* in the metric MSRCC indicates lower error in the Pearson correlation. In both tests, small *p*-values (typically p<0.05) support statistical significance. The results are summarized in Table 3 and they are consistent for both sports datasets. They show that both PairDUG variants yield statistically significant performance improvements that are unlikely to have arisen by chance on both datasets. Only the MSRCC results for Semihard and Coreset sampling may be due to randomness.

### 7.2. Gradient Quality Results

We measure the magnitudes and qualities of the gradient signals to demonstrate the properties of the batch composition of PairDUG’s selected pairs that the Siamese Networks use for training.

We compute four metrics on each method’s gradient updates, i.e., mean gradient norm ${\bar{∥g∥}}_{2}$, gradient diversity ${\bar{D}}_{g}$, the coefficient of variation ${\mathrm{CV}}_{g}$, and the Gradient’s Signal-to-Noise ratio ${\mathrm{SNR}}_{g}$. The experiments were performed on the football dataset with 100,000 training samples, I=500, and Acq=128 selected pairs. We show the mean metrics for the first 1000 batches of the training, which is equal to five epochs.

**Figure 9:**
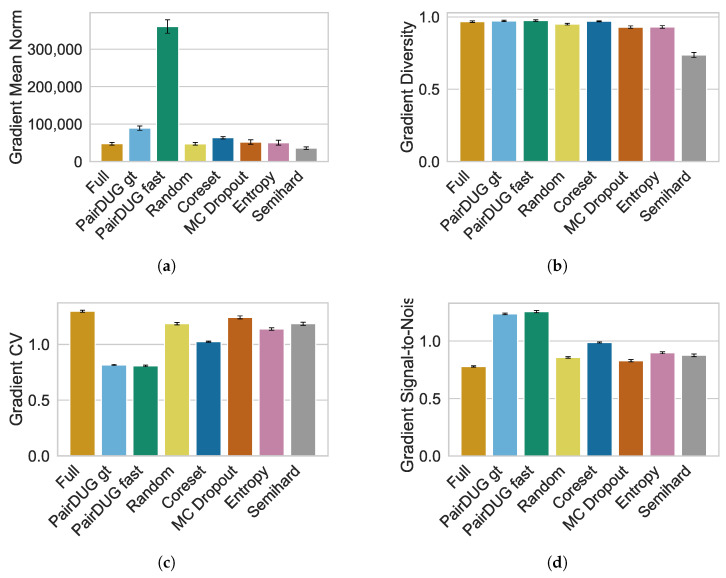
We report the gradient metrics for the football dataset. (**a**) shows the Mean Gradient Norm that measures the typical magnitude of a batch, where larger values suggest stronger updates. (**b**) shows the Gradient Diversity is the average pairwise cosine distance between gradients and captures the information diversity. (**c**) shows the Gradient CV quantifies the relative variability of gradient magnitudes with small values indicating homogeneous gradient magnitudes. (**d**) shows the Gradient SNR indicates consistency in gradient magnitudes, with higher values indicating more consistent gradient signals. Each method produces batches of samples that exhibit different gradient profiles. The PairDUG variants select batches with higher gradient magnitudes than any other sampler, as shown in (**a**). Furthermore, these gradient signals are among the most diverse, as (**b**) shows. (**c**) demonstrates that the batch samples have a highly homogeneous gradient magnitude, and (**d**) confirms this with a high signal-to-noise ratio; both results indicate that PairDUG constructs more informative batches that contain few samples with small gradient updates.

The mean gradient norm ${\bar{∥g∥}}_{2}$ of both PairDUG variants is higher compared to other methods; see Figure 9a. This indicates strong updates, especially for the fast variant. At the same time, the gradient diversity ${\bar{D}}_{g}$ shown in Figure 9b is slightly higher than even what a “simulated full training” would achieve. Batches are also more diverse than those that Random sampling can provide. Only Coreset performs comparably. However, its ${\bar{∥g∥}}_{2}$ is considerably lower than “PairDUG fast” and thus it is less informative. A small gradient coefficient of variation ${\mathrm{CV}}_{g}$ indicates that a batch has lower variability in gradient magnitudes; i.e., both PairDUG variants have high gradient magnitudes that are consistent. In contrast, full training and random sampling are naturally more noisy; see Figure 9c. Finally, the gradient SNR ${\mathrm{SNR}}_{g}$ of both PairPUG variants is higher by more than 0.2 points compared to any other method, and higher by more than 0.4 points than full training; see Figure 9d. These results mirror ${\mathrm{CV}}_{g}$ and indicate that the batch gradients of our proposed method are more coherent.

The evaluation of gradient quality indicates that the subsampled batches of PairDUG capture higher-quality gradient signals, enabling the Siamense Network to learn better representations. The batches have higher gradient magnitudes, which are more consistent and diverse than those from any other method. We also show that PairDUG fast is on par with the more costly PairDUG gt algorithm. However, these results are not direct indicators of downstream performance of the Siamese Network, as there is no statistical correlation between the mean gradient norm and the MAPE or MSRCC. The gradient metrics rather indicate how informative the data is [11]; see also Appendix B for further analysis of training convergence.

### 7.3. Training Speed

We demonstrate that the “PairDUG fast” sampler allows training a Siamese Network of the same quality as full training, but at a fraction of the cost. We report the training speed and MAPE of the model on the test set, with a fixed budget of five epochs for the football dataset, and compare the relative change in MAPE and runtimes. The training computes the optimal Hungarian cost for each training sample, which is the principal cost we aim to reduce with our contribution. We simplify the analysis by proposing a weighted efficiency defined as $E=\frac{M^{\alpha }}{R}$ where $M=\frac{{\mathrm{MAPE}}_{\mathrm{method}}}{{\mathrm{MAPE}}_{\mathrm{full}}}$ and $R=\frac{{\mathrm{Runtime}}_{\mathrm{method}}}{{\mathrm{Runtime}}_{\mathrm{full}}}$. We then control the trade-off between accuracy and runtime using α. The results in Table 4 show that full training takes 9.1 min and the trained model has a MAPE of 5.89. PairDUG fast takes about half the time, at 4.7 min, and keeps a slightly better MAPE of 5.68. The ground-truth-based PairDUG gt even samples batches so that the Siamese Network achieves a MAPE of only 5.5, but training is slightly longer than full training at 9.2 min. Random sampling is faster at 2.6 min, but its MAPE is only 6.46, while Semihard (6.98), MC Dropout (7.45), and Entropy (7.70) score similarly high or worse MAPE at comparable runtimes. Interestingly, Coreset performs better than random with regard to MAPE, but with a runtime about 1 min longer.

We consider preserving low MAPE to be substantially more critical than reducing runtime. However, we still select the *E* value for alpha in a way that rewards both. The sensitivity analysis with α ranging from 3 to 6 confirms that PairDUG fast consistently outperforms all baselines and does not trade off accuracy for speed. Our proposed method performs similarly to or better than full training in this evaluation and cuts the runtime in half. For larger training datasets, such as the simulated full training with 10 million samples for our basketball experiments, the cost savings will be even more pronounced.

Please refer to Appendix B for a visualization of loss curves of the training and validation losses to illustrate training convergence.

### 7.4. Ablation Study

This Section performs ablation studies and sensitivity analysis on the components of our “PairDUG fast” algorithm. These include the method to estimate similarity at lower cost than ground truth in Section 7.4.1, the total budget, i.e., the reduction of the training dataset size, in Section 7.4.2, and the most important hyperparameters in Section 7.4.3.

#### 7.4.1. Trajectory Similarity Approximation

This section compares different methods for the approximation of the trajectory similarity with regard to their retained quality, i.e., Mean Absolute Error (MAE), MAPE, and correlation with ground-truth cost, and their runtime in milliseconds (ms). Any of the evaluated methods can be used in PairDUG fast to calculate the similarity between pairs of trajectories for use with its proxy loss. These are the underlying distances that determine the quality of the selected samples compared to the optimal “PairDUG gt” version, which is practically infeasible as it devolves to the runtime of full training. We evaluate Uniform and Random sub-sampling for the Hungarian algorithm, Dynamic Time Warping (DTW) [42], and several dynamic sampling heuristics:

- Density treats each time step as a point in spatial space (e.g., the mean position of all players). Then it clusters those points into *n* keypoints, i.e., spatial regions, and uses the time steps closest to cluster centers as representative keypoints. This ensures dense regions (where trajectories revisit similar positions) are represented proportionally, while avoiding redundant samples. However, it disregards the temporal spread of positions.
- Change Detection spreads keypoints such that each one corresponds to roughly equal cumulative motion change. It first computes the cumulative motion magnitude and distributes the keypoints by the amount of change. This heuristic adapts to bursty motion.
- Spatiotemporal Diversity selects keypoints that maximize coverage of the trajectory in both spatial and temporal dimensions. It treats each scene as a spatiotemporal vector (x,y,n), where *n* is the number of keypoints, and performs k-means clustering on this joint space.

Table 5 shows errors between the Hungarian algorithm’s optimal solutions and the estimates based on sub-sampling to (5,10,20,30,40) keypoints. Uniform sampling performs best for n∈(10,20,30,40) in any metric and dataset. It is only surpassed by Spatiotemporal Diversity and Diversity sampling for n=5 on the basketball dataset, albeit with runtime costs two orders of magnitudes higher. Uniform sampling appears to outperform other heuristics, at least in part, because they exhibit negative biases that do not generalize to the very diverse data and scenes that basketball and American football represent.

We conclude that Uniform sampling is the most suitable method for use with PairDUG fast from all tested samplers, as it delivers the highest quality approximation at the lowest runtime cost. Furthermore, it adapts to two different datasets better than any other method and has the lowest approximation errors. It appears that the optimal number of keypoints is 20 based on the MAE and MAPE of the estimated and ground-truth pairwise distances. In the downstream task of model training, we furthermore observe that the quality loss of the approximated PairDUG fast compared to PairDUG gt is minimal while the fast variant halves the training time; see Section 7.1.

#### 7.4.2. Budget Performance Impact

We report results for different budget sizes (training dataset sizes) by setting the sizes of the subset *I* to different values. For different-sized *I*, the absolute number of pairs seen during an epoch is reduced. However, during each epoch, other pairs may be selected for training.

**Figure 10:**
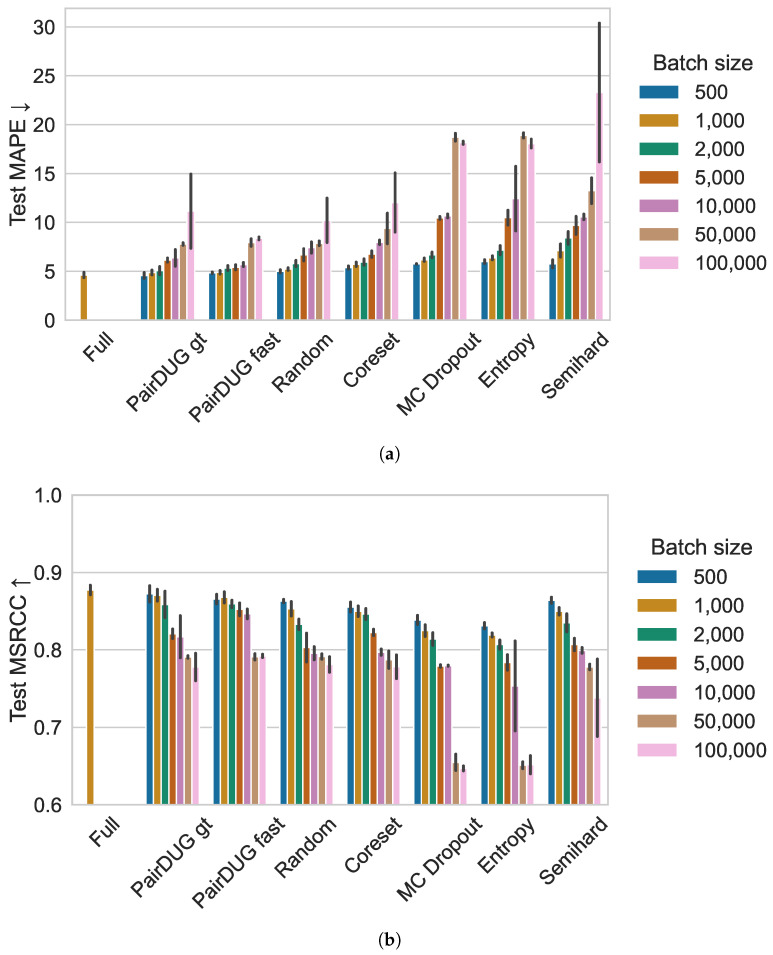
This figure shows the results of the sensitivity analysis using the American football data for different sizes of the subset *I* for all samplers. (**a**) reports the MAPE scores for different batch sizes and (**b**) reports the corresponding MSCRR scores. The small |I|=500 permits the samplers to select about 25 k samples per epoch by sequentially sampling Acq=128 from a window of 500. Furthermore, more acquisitions of smaller size also permit the Active Learner to select more appropriate samples at different points of the task model learner’s state. The quality of the learned embedding drops significantly with only 128 training samples at |I|= 100,000. Still, PairDUG variants are the most stable even for larger batch sizes.

We show the MAPE and MSCRR scores for each sampler in Figure 10, and report the subset sizes |I|∈ (500, 1000, 2000, 5000, 10,000, 50,000, 100,000). This amounts to (25,600, 12,800, 6400, 2560, 1280, 256, 128) selected pairs per epoch; i.e., samplers may select other pairs in each epoch, enriching the training. Full training is the baseline for maximal performance, and random sampling is the benchmark. The figures show that our PairDUG variants outperform the baselines across most subset sizes and yield more stable, reliable training results with lower variance. With smaller subset sizes, this becomes a significant problem to mitigate, as samplers show very high variance for |I|> 10,000.

The performance variation with different subset sizes is primarily determined by the selection biases of the methods that are either sampling with a certain level of representativeness (PairDUG, Coreset) or that do not account for any similarity within the selected pairs (MC Dropout, Entropy, Semihard). The resulting performance variations for larger subset sizes clearly show the well-known problem of mode collapse that uncertainty-based sampling also suffers from: without any constraint enforcing diversity, samplers may over-sample the least certain samples or anomalies [31]. Smaller subset sizes may exhibit a lower performance variation simply because they contain fewer samples from the same (uncertain or anomalous) regions of the dataset than larger subsets [31].

#### 7.4.3. Hyperparameter Ablations

This section ablates the most important hyperparameters of PairDUG. These are the optimizers’ Learning Rate, the Neural Network’s Dropout Rate, and Embedding dimensionality (number of neurons in the penultimate layer), as well as the acquisition size per step, i.e., the value of Acq in PairDUG’s k-means++ clustering of gradient embeddings to select Acq samples for training. We use the following defaults: Dropout Rate of 0.3, Embedding dimensionality of 64, and a Learning Rate of 0.001. For the ablations on Acq, we set the size of the subsampled data to 2000 pairs and default Acq to 128 centroids (6400 pairs in training per epoch).

Table 6 presents the results. These indicate that PairDUG is robust with regard to changes in the Dropout Rate and the Embedding dimensionality. The differences in either metric are minimal and within the range of noise. In contrast, the learning Rates 0.0001 and 0.001 lead to significantly lower MAPE and higher MSRCC, which are also more stable. Finally, varying the parameter Acq to adjust the number of centroids in the k-means++ clustering of PairDUG fast shows a clear trend: larger Acq leads to better performance, as more samples are used for training. Directly comparing absolute budget sizes, setting Acq=128 and sampling from |I|=500 available pairs for a total of 200 steps (refer to Figure 10 with batch size of |I|=500) leads to higher scores compared to setting Acq=512 and sampling from a window of |I|=2000 for a total of only 20 steps. This is due to the cumulative learning effect often observed in Active Learning [31]; i.e., with more and smaller steps, the sample selection by PairDUG becomes more informed at each step.

Overall, the results indicate that our proposed method can be trained robustly across a wide range of hyperparameters. Our default parameters strike a good balance between training convergence and stability, with the selection of the parameter Acq and the window size *I* most useful for adjusting training time and expected performance, balancing efficiency and total training time.

## 8. Discussion

**RQ**_1_: Can the required compute time for training a Siamese Network be reduced while maintaining similar retrieval quality?

Our evaluation shows that reducing training time is possible. In fact, PairDUG fast cuts training time roughly in half while maintaining high retrieval quality, as shown by MAPE and MSCRR. This property is shown for different pruning rates of the training dataset in the ablation studies, where we evaluate different subset sizes. In contrast, other methods entail significant trade-offs. There may be faster sampling methods. However, they produce worse representations, leading to higher MAPE and lower retrieval quality. In fact, random sampling remains a strong baseline that other samplers besides PairDUG cannot consistently beat.

**RQ**_2_: Does active sampling retain a higher retrieval quality compared to other baselines?

Our experiments confirm that active samplers can outperform random sampling in maintaining higher retrieval quality. However, the method must be carefully designed. Our proposed PairDUG sampler combines uncertain and diverse gradients [11] and thus achieves the best scores from all samplers. In comparison, only Coreset [17] sometimes beats the random baseline by selecting diverse batches to train the Siamese Network. Uncertainty-based methods such as MC Dropout and Entropy fail to win against the Semihard sampling method and are not suitable active samplers for the sports scene retrieval application.

**RQ**_3_: What explanations for the active samplers’ effects on learning are there?

We investigate the batch composition with respect to its gradient signals that are crucial for learning. Our PairDUG samplers specifically construct batches with high gradient magnitudes of diverse nature. Furthermore, the gradient signals are consistent, which enables faster training. The gradients also represent higher information value because the Siamese Network requires fewer samples. In contrast, a randomly sampled dataset has less informative samples and more noisy gradients with regard to consistent signals, which are less well suited for learning from fewer samples. Similarly to our method, but to a lesser extent, Coreset benefits from diverse sampling, but has a lower Signal-to-Noise ratio, i.e., worse gradient consistency, resulting in worse retrieval quality.

## 9. Conclusions

Search and retrieval of interesting plays from large datasets of unstructured trajectories is a key problem for sports analytics and other fields with large amounts of positional data. The usual training of Siamese Networks to learn a lower-dimensional representation is costly due to the combinatorial nature of pairwise comparisons and thus employs subsampling that sacrifices quality for speed.

This work adapts and extends methods that select an informative subset from the Active Learning literature to reduce the combinatorial complexity of pairwise similarity learning, such as uncertainty-, diversity-, and gradient-based samplers.

Our proposed PairDUG fast sampler retains the retrieval quality of full training but cuts training time in half by sampling diverse and informative pairs using the Neural Network’s gradient signals, thereby beating random subsampling and other heuristics by a large margin. We analyze the PairDUG fast variant’s estimation error due to its keypoint sub-sampling to determine an error bound, which we show to be practically negligible compared with natural gradient variance within a batch. The experimental results, in addition, confirm that the fast variant performs equally as well as the ground-truth version. Furthermore, we analyze the gradient quality of their selected samples relative to the baselines and demonstrate their high magnitude, diversity, and stability.

Our results show good generalization across the large-scale sports datasets, basketball and American football, and robustness to their hyperparameters. Future work may transfer the sampler to other datasets and domains, as well as to other data mining challenges beyond pairwise distance learning.

Future avenues of research may extend PairDUG with optimized implementations that better utilize GPU-parallelism or make use of alternatives to the Hungarian algorithm, such as the Jonker–Volgenant or Auction algorithms, to increase training efficiency, or develop end-to-end data pruning techniques.

## Figures and Tables

**Table 1:** Notation used throughout this work.

Symbol	Meaning
x	Trajectory matrix of a single entity, $x\in R^{S\times T}$
$x_{s,t}$	Coordinate value at spatial dimension *s* and time *t*
*S*	Spatial dimension (e.g., S=2 for 2D positional tracking)
*T*	Temporal length of the trajectory in frames
X	Scene tensor containing all entities’ trajectories, $X\in R^{N\times S\times T}$
*N*	Number of tracked entities (players and ball) in a scene
$X_{1},\;X_{2}$	Two scenes compared pairwise
$x_{n},\;x_{m}$	Individual trajectories from $X_{1}$ and $X_{2}$
*t*	Temporal index
$d(x,x^{\prime })$	Distance between two trajectories
$d(X_{1},X_{2})$	Scene-to-scene distance after optimal assignment
$C_{jk}$	Cost matrix entry between trajectory *j* and *k*
${\cos t}_{\mathrm{gt}}$	Ground-truth optimal assignment cost
${\cos t}_{\mathrm{fast}}$	Approximated assignment cost using keypoint sub sampling
$n_{\mathrm{keypoints}}$	Number of sampled time indices for approximation
ε	Approximation error between fast and ground-truth cost
$f_{\theta }\left(X\right)$	Embedding function (Siamese branch) with parameters θ
$e_{1},e_{2}$	Embeddings of two scenes, defined as $e_{i}=f_{\theta }\left(X_{i}\right)$
$d(e_{1},e_{2})$	Distance between embeddings
δ	Gradient magnitude proxy = derivative of squared error loss
$g_{i}$	Gradient vector of sample *i* used in gradient metrics.
${\tilde{g}}_{i}$	Normalized gradient vector, used for cosine similarity in diversity metric
$\theta_{\mathrm{emb}}$	Parameters of the embedding layer, used in gradient embedding computation
L	Loss function
λ	Weight decay (regularization coefficient), used in optimization
η	Learning rate, hyperparameter with default $10^{-3}$
${\bar{∥g∥}}_{2}$	Mean gradient norm across batch, indicating update magnitude
${\bar{D}}_{g}$	Gradient diversity, the average pairwise cosine distance
${\mathrm{CV}}_{g}$	Coefficient of variation of gradient norms
${\mathrm{SNR}}_{g}$	Gradient signal-to-noise ratio
*I*	Subset of candidate pairs per iteration or “pool subset”
Acq	Number of pairs actively selected per step from *I*
*P*	Number of stochastic forward passes
$\sigma^{2}$	Predictive variance
*H*	Entropy of predictive distribution
$E,\hat{E}$	Distance lists for rank correlation calculation
*r*	Spearman rank correlation coefficient

**Table 2:** The dimensionality of each dataset’s scenes is defined by the number of entities with trajectories, i.e., players and ball, as well as the length of each scene. We preprocess the scenes and split them into training, validation, and test sets, with the basketball data being a magnitude larger in scale. It is crucial to point out that the 10 M generated pairs of basketball scenes are only 0.2% of the 5 billion possible combinations, and the 100 k of American football amount to 2%. With larger datasets, the combinatorial complexity becomes even larger, and active selection becomes more important.

Sport	Entities	Length	Scene Splits	Pairs
Basketball	11	150 (6 s@25 Hz)	99,327/12,360/12,366	10 M/500 k/500 k
American Football	23	50 (5 s@10 Hz)	3106/384/386	100 k/100 k/100 k

**Table 3:** Two-sample *t*-tests comparing each method with Random sampling for basketball and American football. Columns are grouped by evaluation metric.

Method	Test MAPE ↓				Test MSRCC ↑			
	Basketball		Am. Football		Basketball		Am. Football	
	*t*	*p*	*t*	*p*	*t*	*p*	*t*	*p*
Full	−4.0	6.9 $\times \;10^{-3}$	−4.5	4.4 $\times \;10^{-3}$	3.9	1.6 $\times \;10^{-3}$	4.9	1.9 $\times \;10^{-3}$
PairDUG gt	−2.6	3.4 $\times \;10^{-2}$	−2.8	3.1 $\times \;10^{-2}$	3.0	2.1 $\times \;10^{-2}$	3.3	1.2 $\times \;10^{-2}$
PairDUG fast	−2.9	2.0 $\times \;10^{-2}$	−3.3	1.3 $\times \;10^{-2}$	2.6	3.0 $\times \;10^{-2}$	2.8	2.5 $\times \;10^{-2}$
Coreset	3.8	7.6 $\times \;10^{-3}$	4.2	5.0 $\times \;10^{-3}$	−0.9	3.5 $\times \;10^{-1}$	−0.6	5.5 $\times \;10^{-1}$
Semihard	5.4	4.5 $\times \;10^{-3}$	6.2	2.8 $\times \;10^{-3}$	−1.0	5.0 $\times \;10^{-1}$	−0.7	5.1 $\times \;10^{-1}$
MC Dropout	7.7	3.5 $\times \;10^{-4}$	10.0	1.3 $\times \;10^{-5}$	−4.9	1.2 $\times \;10^{-3}$	−5.3	8.6 $\times \;10^{-4}$
Entropy	8.3	2.1 $\times \;10^{-4}$	10.8	1.3 $\times \;10^{-5}$	−5.3	9.1 $\times \;10^{-4}$	−7.8	7.7 $\times \;10^{-4}$

**Table 4:** Duration in minutes for a training of 5 epochs and MAPE on the testset. Calculations include computing ground-truth distances using the Hungarian algorithm. MAPE Weighted Efficiency across different α values shows PairDUG fast performs best. The duration does not include data preprocessing and only loads data from fast NVMe SSD storage. Furthermore, full training is simulated with only 2% for the possible scene pairs.

Method	Duration	MAPE	α=3	α=4	α=5	α=6
PairDUG fast	4.7	5.68	1.96	2.09	2.25	2.42
Coreset	3.9	6.29	1.91	1.81	1.72	1.63
Random	2.6	6.46	1.77	1.65	1.55	1.45
PairDUG gt	9.2	5.55	1.19	1.19	1.20	1.21
Semihard	3.5	6.98	1.13	1.09	1.06	1.03
Full (simulated)	9.1	5.89	1.00	1.00	1.00	1.00
MC Dropout	2.7	7.45	1.17	1.04	0.91	0.81
Entropy	2.7	7.70	1.12	0.96	0.82	0.71

**Table 5:** Comparison of trajectory alignment methods on basketball and American football datasets, for 1.000 pairs per dataset. Lower MAE/MAPE and higher correlation (Corr) with ground truth are better. Uniform sampling of n∈(20,30) keypoints appears to be a good trade-off between runtime and quality. Other heuristics underperform or are computationally too expensive.

Method (*n* Keypoints)	Basketball Dataset				American Football Dataset			
	MAE	MAPE	Corr	Time (ms)	MAE	MAPE	Corr	Time (ms)
Hungarian	0.00	0.0000	1.0000	0.07	0.00	0.0000	1.0000	0.11
DTW	284.02	0.7872	0.9866	60.00	754.53	0.8717	0.9843	6.79
Uniform (5)	13.38	0.0462	0.9971	0.06	6.15	0.0100	0.9995	0.07
Uniform (10)	6.10	0.0209	0.9994	0.04	2.93	0.0046	0.9999	0.06
Uniform (20)	3.01	0.0102	0.9997	0.04	1.89	0.0027	0.99997	0.06
Uniform (30)	1.57	0.0056	0.99996	0.04	1.75	0.0025	0.99997	0.07
Uniform (40)	1.31	0.0045	0.99997	0.06	1.84	0.0026	0.99997	0.08
Random (5)	30.13	0.0937	0.9687	0.07	22.86	0.0352	0.9914	0.09
Random (10)	20.51	0.0659	0.9861	0.06	13.45	0.0215	0.9982	0.08
Random (20)	13.58	0.0419	0.9944	0.06	9.19	0.0131	0.9993	0.08
Random (30)	12.10	0.0392	0.9955	0.06	7.07	0.0117	0.9993	0.09
Random (40)	9.80	0.0308	0.9969	0.06	2.76	0.0044	0.99995	0.10
Change Detection (5)	22.20	0.0699	0.9863	0.07	16.79	0.0254	0.9941	0.08
Change Detection (10)	19.30	0.0587	0.9878	0.05	16.28	0.0255	0.9962	0.07
Change Detection (20)	19.29	0.0586	0.9887	0.06	16.82	0.0261	0.9958	0.08
Change Detection (30)	19.06	0.0580	0.9896	0.06	16.99	0.0263	0.9957	0.09
Change Detection (40)	19.11	0.0585	0.9898	0.06	17.54	0.0269	0.9950	0.09
Density (5)	12.71	0.0421	0.9955	3.56	9.08	0.0144	0.9987	2.85
Density (10)	11.24	0.0377	0.9968	4.65	6.41	0.0110	0.9998	3.88
Density (20)	9.99	0.0319	0.9972	7.09	6.49	0.0112	0.9996	6.52
Density (30)	9.19	0.0294	0.9978	9.96	11.47	0.0176	0.9993	9.17
Density (40)	9.34	0.0300	0.9976	12.32	9.92	0.0148	0.9991	12.04
Spatiotemporal Diversity (5)	12.71	0.0421	0.9955	2.88	9.07	0.0144	0.9987	2.22
Spatiotemporal Diversity (10)	11.24	0.0377	0.9968	3.91	6.40	0.0109	0.9998	3.15
Spatiotemporal Diversity (20)	10.04	0.0320	0.9972	6.01	6.68	0.0114	0.9995	5.34
Spatiotemporal Diversity (30)	9.18	0.0294	0.9978	8.28	11.68	0.0180	0.9993	7.62
Spatiotemporal Diversity (40)	9.28	0.0299	0.9976	10.47	10.04	0.0151	0.9991	10.14

**Table 6:** Summary of MAPE and MSRCC across hyperparameters for the American football dataset.

Hyperparameter	Value	MAPE (mean ± std)	MSRCC (mean ± std)
Dropout Rate	0.5	5.002 ± 0.139	0.864 ± 0.005
Dropout Rate	0.3	5.012 ± 0.251	0.865 ± 0.005
Dropout Rate	0.1	4.991 ± 0.129	0.865 ± 0.005
Embedding Dim	1024	4.902 ± 0.271	0.867 ± 0.007
Embedding Dim	128	5.029 ± 0.252	0.865 ± 0.007
Embedding Dim	64	5.012 ± 0.251	0.865 ± 0.005
Embedding Dim	32	4.914 ± 0.306	0.869 ± 0.007
Acq centroids	512	4.762 ± 0.097	0.870 ± 0.005
Acq centroids	256	4.862 ± 0.109	0.867 ± 0.005
Acq centroids	128	5.012 ± 0.251	0.865 ± 0.005
Acq centroids	64	5.274 ± 0.214	0.859 ± 0.003
Acq centroids	32	5.428 ± 0.409	0.852 ± 0.002
Learning Rate	0.01	6.807 ± 0.753	0.832 ± 0.025
Learning Rate	0.001	5.012 ± 0.251	0.865 ± 0.005
Learning Rate	0.0001	4.922 ± 0.157	0.869 ± 0.005

**Figure A1:**
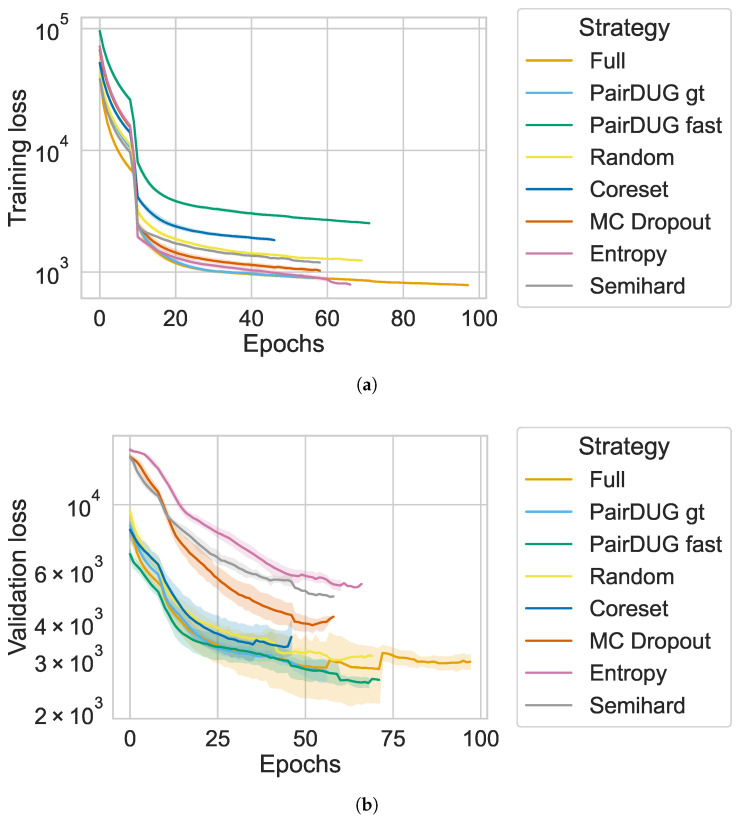
Training and validation loss curves for the American football dataset. Each line corresponds to a different sampling strategy. Experiments were conducted five times, and training converged at different numbers of epochs with early stopping. The plots show how quickly and stably each method minimizes loss during training and how well it generalizes on validation data. (**a**) Training loss curves illustrate that each strategy exhibits different convergence behavior. Both PairDUG variants construct batches with a higher average training loss. (**b**) Validation loss curves illustrate generalization performance with lower validation losses suggesting better generalization. Both PairDUG variants exhibit low validation losses, comparable to full training but with earlier convergence with regard to the number of epochs.

## Data Availability

The data presented in this study is available on Github at https://github.com/crispchris/Pairwise-Diverse-and-Uncertain-Gradient-Sampling-for-Similarity-Retrieval (accessed on 10 November 2025). These data were derived from the following resources available in the public domain: https://github.com/linouk23/NBA-Player-Movements; https://github.com/asonty/ngs_highlights.

## Abbreviations

The following abbreviations are used in this manuscript:

PairDUG	Pairwise Diverse and Uncertain Gradients
BADGE	Deep Batch Active Learning by Diverse, Uncertain Gradient Lower Bounds
MAPE	Mean Absolute Percentage Error
MSCRR	Top-N Mean Spearman Correlation Coefficient
MAE	Mean Absolute Error
MC Dropout	Monte-Carlo Dropout

## Appendix A. Computational Complexity

This appendix discusses the asymptotic computational complexity of a **Full training** of the contributed methods **PairDUG fast** and **PairDUG gt**, as well as the baselines **Random** and **CoreSet** sampling. Please see Table A1 for a concise overview. The remainder of this section analyses the complexities of the methods.

The analysis follows our notation and uses *N* for the number of scenes of length *T*, Acq for the number of pairs selected per acquisition, and *d* for the size of the embedding dimension. Furthermore, $C_{Hungarian}$ denotes the cost of the Hungarian algorithm, which is $O(n^{3})$ in the number of trajectories in a scene.

- Full training generates all pairs $\frac{N(N-1)}{2}\approx O\left(N^{2}\right)$ for a dataset; each requires one Hungarian computation of $O(n^{3})$, resulting in full costs of $O\left(N^{2}\;\cdot \;C_{Hungarian}\right)=O\left(N^{2}n^{3}\right)$.
- Random sampling selects Acq instead of *N* pairs per epoch, each requiring a Hungarian cost, resulting in a total cost of $O(Acq\;\cdot \;n^{3})$.
- CoreSet sampling requires a forward pass of Acq samples of dimensionality *d* with O(Acq·d), calculates a distance matrix for its greedy K-Center of $O(Acq^{2}\;\cdot \;d)$ and greedily iterates over the Acq points with $O(Acq^{2}\;\cdot \;d)$. This results in an overall cost of $O(Acq\;\cdot \;n^{3}+Acq^{2}\;\cdot \;d)$, where the second term dominates with growing Acq.
- PairDUG gt consists of four steps. First, the embedding computation in the forward pass has an asymptotic computational complexity of O(Acq·d). Then, ground-truth Hungarian distances are computed for each of the Acq pairs with $O(Acq\;\cdot \;n^{3})$, followed by the gradient embedding construction with O(Acq·d). Finally, K-means++ clustering over these Acq gradient embedding vectors has an associated cost of $O(Acq\;\cdot \;d\;\cdot \;n_{iter})$ where $n_{iter}$ is the number of iterations required by K-means++ for convergence, typically very few and considered constant for this analysis. Therefore, the total cost is $O(Acq\;\cdot \;n^{3}+Acq\;\cdot \;d\;\cdot \;n_{iter})$. With $d\ll n^{3}$, the Hungarian cost dominates the cost of PairDUG gt, dwarfing the cost of clustering.
- PairDUG fast aims to reduce the cost of the Hungarian algorithm by using keypoint sub-sampling to $n_{pts}$, reducing the cost to $O(Acq\;\cdot \;n_{pts}^{3})$. In addition, it still has costs of O(Acq·d) for the embedding computation and a term for K-means++ clustering of $O(Acq\;\cdot \;d\;\cdot \;n_{iter})$. Hence, the total cost is $O(Acq\;\cdot \;n_{pts}^{3}+Acq\;\cdot \;d\;\cdot \;n_{iter})$. Since $n_{pts}\ll n$, the first term is reduced by factor ${\left(\frac{n_{pts}}{n}\right)}^{3}$. With $n_{pts}=20$ and n=150, this is equivalent to a 27× speedup. The longer the trajectory data, the greater the potential speedup. The clustering term may become comparable to the cheaper Hungarian algorithm’s cost, but is still linear in Acq and *d*.

Both PairDUG variants use K-means++ clustering over the Acq gradient embeddings of dimensionality *d*, adding $O(Acq\;\cdot \;d\;\cdot \;n_{iter})$ with $n_{iter}$ iterations necessary for clustering to converge. For the variant “gt”, clustering is negligibly cheap compared to the complexity of the Hungarian of $O(Acq\;\cdot \;n^{3})$. For the variant “fast”, the approximated distance computation with $n_{pts}\ll n$ is reduced to $O(Acq\;\cdot \;n_{pts}^{3})$, and clustering may become dominant. However, the complexity $O(Acq\;\cdot \;d\;\cdot \;n_{iter})$ still scales only linearly with Acq and *d* and is sub-quadratic.

**Table A1:** Asymptotic computational complexity of the compared strategies. *N* = number of scenes, Acq = number of sampled pairs per epoch, *d* = embedding dimension, *n* = number of trajectories per scene, $n_{\mathrm{pts}}$ = number of temporal sub-samples, $n_{\mathrm{iter}}$ = K-means++ iterations.

Method	Distance	Clustering/Sampling	Total
Full Training	$O(N^{2}n^{3})$	–	$O(N^{2}n^{3})$
Random	$O(Acq\;\cdot \;n^{3})$	–	$O(Acq\;\cdot \;n^{3})$
CoreSet	$O(Acq\;\cdot \;n^{3})$	$O(Acq^{2}\;\cdot \;d)$	$O(Acq\;\cdot \;n^{3}+Acq^{2}\;\cdot \;d)$
PairDUG-gt	$O(Acq\;\cdot \;n^{3})$	$O(Acq\;\cdot \;d\;\cdot \;n_{\mathrm{iter}})$	$O(Acq\;\cdot \;n^{3}+Acq\;\cdot \;d\;\cdot \;n_{\mathrm{iter}})$
PairDUG-fast	$O(Acq\;\cdot \;n_{\mathrm{pts}}^{3})$	$O(Acq\;\cdot \;d\;\cdot \;n_{\mathrm{iter}})$	$O(Acq\;\cdot \;n_{\mathrm{pts}}^{3}+Acq\;\cdot \;d\;\cdot \;n_{\mathrm{iter}})$

## Appendix B. Loss Curves

This appendix discusses the loss curves for all evaluated methods in Figure A1 to illustrate the convergence speed of the Siamese Network’s training. The plots show that the PairDUG variants construct batches that cause substantial training losses on the one hand and small validation losses on the other hand. Crucially, lower validation loss suggests better generalization.
